# An Inquiry Illustrating the Nature of Tuberculated Accretions of the Serous Membranes; and the Origin of Tubercles and Tumours in Different Textures of the Body; with Engravings

**Published:** 1819-10-01

**Authors:** 


Medico-Chirurgical Journal;

OR,

Quarterly Register

OF

MEDICAL AND SURGICAL SCIENCE.

No. 6.] OCTOBER 1, 1819. [VOL. II.

### First Department.

### BIBLIOLOGY;

OR,

ANALYTICAL REVIEWS OF MEDICAL PUBLICATIONS,

*Interwoven with*

ORIGINAL OBSERVATIONS, AND PRACTICAL RESEARCHES.

Nec tibi quid liceat sed quid fecisse decebit

Occurrat mentemque domat respectus honesti. CLAUD.

### I.

*An Inquiry illustrating the Nature of tuberculated Accretions of the serous Membranes; and the Origin of Tubercles and Tumours in different Textures of the Body; with Engravings.* By JOHN BARON, M.D. Physician to the General Infirmary at Gloucester. One Volume, Octavo, 308 pages, and Five Plates. London, 1819.

A NOTED teacher in the British Metropolis is accustomed to observe to his pupils, that medical men, of the present day, are too apt to turn old doctrines and hypotheses upside down, give them new names, and then come out with great discoveries. We have also heard it said lately, that the same remark is applicable to certain diseases themselves, which, on being more accurately examined by modern pathologists, have acquired new designations, and been considered additions to the long and melancholy catalogue of human infirmities. But for our own parts, we are by no means so fastidious on these points, being quite of Dr. Baron's way of thinking—namely, that "successive speculations on any subject imply, for the most part, successive approximations to truth, as no false opinion can be destroyed but by the accession of information, whereby its falsity is evinced. The theories, therefore, which an improved philosophy sanctions are the excitements to further inquiry; the bonds which hold together the disjointed elements of knowledge; not impeding the march of truth, but preparing the way for her ultimate triumph." Speculations, however, become pernicious when they are founded on the mere conjectures of "daring spirits," instead of facts and accurate observations. We are hardly yet freed from the spell of a late presumptuous and speculating genius, whose splendid illusions dazzled the mental optics of all Europe, and caused more havoc among the human race than the scourge of war itself.

We agree with our author, that the post mortem appearances, in diseased bodies, have been much more minutely and faithfully registered, than the symptoms which marked the successive changes of structure during life. No man can peruse Bonetus and Lieutaud, without feeling the force of this remark; and even Morgagni, the accurate and honest Morgagni, gives us but very lame and imperfect histories of most of those cases, whose dissections are recorded in his valuable but neglected work.

It has been the object of our author to connect the symptoms with the functional and organic changes induced by disease; and for this purpose he has compared the experience of his predecessors with his own observations. What he at first thought an affection peculiar to membranes of the serous class, and explicable by the commonly received physiological and pathological doctrines, he now finds to be connected with the whole tribe of cachectic diseases, and various other disorganizations in different textures of the body.

The work is divided into two parts; the first, on the peritonæum; the second, on the pleura.

1. *History—Cases—Dissections.* Acute or purely inflammatory affections of the serous membranes have been well described; but the chronic changes of structure are very obscure, and have not hitherto been accurately traced. The disease under consideration is almost uniformly fatal; nor is it, Dr. Baron thinks, of rare occurrence. He has seen it in persons of all ages; though more in females than males. It comes on, in general, with tenderness and distention of the abdomen, accompanied with nausea and vomiting. The bowels, for the most part, are costive, both before and after the attack; but they are frequently relaxed. In the *progress* of the disorder the bowels become more and more irregular in their action. The tenderness and swelling increase; the appetite fails; the features sharpen and contract; the countenance becomes pale or sallow; the lips parched and skinny; the tongue sometimes of a bright colour, as in diabetes; at others, covered with a thick whitish mucus. The flesh and strength decay more rapidly than in any other disease; the skin, except towards the last stage, is commonly dry and scaly; the urine scanty, and frequently turbid; cough, if not from the beginning, is now apt to occur, if the disease spreads from the abdominal to the thoracic viscera.

"The feet sometimes swell towards the conclusion of the disease, but I have often seen the swelling confined to one leg and thigh. At this period, if the examination of the abdomen be made with due care, it will be found to communicate to the touch the feeling occasioned by a solid tumour; the integuments and muscles not rolling upon the contained parts as in a state of health. But in some cases, where effusion is conjoined with the original and more important disease, a sense of fluctuation may be discovered.

"Very frequently the patients complain of a distressing feeling of a "*broiling heat*" at the stomach, the discharge of a tough, ropy, phlegm from the mouth, constant nausea, with violent retching and vomiting; and in two cases, the matter brought up during several days before death was stercoraceous." P. 20.

Although the appetite fails, the thirst is insatiable in the progress of the disease, even when the fluid swallowed occasions great distress. When sustenance is taken it is either speedily rejected, or causes indescribable uneasiness. The patient rolls about in all directions, seeking, in vain, for some point where he may repose. Every action of the stomach and intestines comes to be performed with great pain. The passage of flatus upwards or downwards, the movements which take place previous to the evacuation of the bowels, all give rise to much suffering.

"At times the pain is sharp and transient, at others, it is heavy and obtuse. But a sense of weight is seldom absent; and it is more felt after vomiting or purging than before. One patient (an infant) in allusion to this symptom, used to put his hand on the abdomen, and exclaim "Oh! so heavy!" Another said that his bowels felt as if they were "tied up in a napkin." At another time he said—"they seemed to be in a mass;" and at a third, he declared that if he had "a shot attached to every convolution of his intestines he could not suffer more than he did." P. 22.

The above expressions were used by a medical man, as will be seen hereafter. The *post mortem* appearances illustrate the symptoms during life. The stomach and intestines cannot perform their functions, and the necessary aliment acts as a foreign body, occasioning dreadful distress. The source of external supply being thus cut off, the system feeds upon itself, as long as matter sufficient can be taken up by the absorbents to keep the vital functions in action. The change of structure is universal accretion of the peritonæum, binding all the contained and containing parts of the abdomen into one confused mass. The membrane becomes much thickened; loses its smooth and shining appearance, and is studded throughout with innumerable tubercles of various sizes. These are sometimes single, sometimes in clusters, pervading also the mesentery and omentum, while the lymphatic glands are frequently found enlarged and diseased.

Case I. The first patient which our author met with, labouring under this disease, was a girl 17 years old, who, when admitted into the Infirmary, was affected with a hard, tense swelling of the whole abdomen, painful on pressure, with sharp, pallid, anxious countenance; weak emaciated frame; cough, and copious expectoration; tongue of a bright red colour; thirst urgent; pulse quick and feeble; appetite bad; bowels loose. The complaint was said to be of only four weeks duration. Squills and calomel twice a-day, with aloes and iron seemed, at first, to effect a slight reduction of the abdominal swelling; but the emaciation went on; the debility increased; the feet swelled; there were nocturnal perspirations; constant nausea and vomiting, with dreadful anxiety and restlessness, till death terminated her sufferings in less than three months.

*Dissection.* The whole abdominal viscera were adherent, and appeared, *en masse.* They could not be distinguished till the thickened layers of the peritonæum were torn from their adhesions. The intestines could not possibly be unfolded or traced. The alimentary tube itself had undergone a remarkable change. It was found deprived of its healthy texture; had lost its tenacity, and appeared like a transparent membrane which had been macerated in water. It tore on the slightest violence being applied to it. The mesentery and glands were diseased; the liver enlarged, and had lost its natural texture. A lumbricus was discovered in one of the biliary ducts, measuring full six inches in length, and alive at the time of examination. In the pleura some changes analogous to those in the peritonæum. Universal adhesion on the left side; diaphragm studded with small granulated masses; in the left lobe of the lungs tubercles in a state of suppuration.

We shall pass over several cases bearing a great similitude to that just related, in order to present some particulars of the following.

Case II. W. F. Shrapnell, Esq. surgeon, after serving some arduous campaigns in Ireland, as a military surgeon, in 1816, and suffering from dysentery, became debilitated, and affected with sick head-ache, a malady to which he had been long subject. In January 1817, he first consulted Dr. Baron by letter. He then complained chiefly of pain in the right hypochondrium, diverging to the scrobiculus cordis and to the spine; some tenderness on pressure of the left lobe of the liver. He pursued a slight mercurial course for some weeks, without benefit, when it was dropped. He now very imprudently put his constitution to the test, by walking twenty-four miles in one day. He was taken up by Dr. Jenner towards the close of this journey; but the motion of the carriage gave him pain; chiefly about the right hypochondrium. He could find no easy posture. From this time his complaints increased rapidly. He felt as though his internal organs were compressed with a bandage.

On the 15th of May, Dr. Baron first saw the patient, who had now a sharp pain, increased by pressure, in the right side; the belly enlarged and tender; there was great languor and distress, with a peculiarly anxious countenance; sense of weight in the abdomen; irregularity of bowels; inappetency; urgent thirst; much uneasiness after swallowing any thing; pulse nearly natural. He had been bled several times from the arm, and the abdomen leeched repeatedly. The blood was inflamed, and relief followed each bleeding. It was determined to carry the general and local bleeding still farther. During the next four days he was bled from the arm and leeched three or four times; always with temporary relief: but still the disease did not seem to yield. The general swelling of the abdomen increased. Pressure on every point of the surface conveyed the idea of a solid mass; the bowels were kept sufficiently open, but the stools were passed with difficulty.

After the depleting system had been carried as far as prudence would permit, he was occasionally purged with calomel and colocynth, while it was attempted to mitigate his sufferings by the anodyne extracts. The warm bath; mercurial frictions over the abdomen; artificial Cheltenham waters, &c. were tried, but only gave momentary relief. At length a sharp ridge could be felt in the course of the arch of the colon, under which the fingers could be pushed; and soon afterwards there appeared decisive proofs that a fluid was effused into the abdomen. In two days from this time, viz. on the 6th of June, paracentesis abdominis was performed by Mr. Fry, and great relief was experienced during the flow from the trochar. The ridge before felt, now projected an irregular tumour. Although two gallons of yellow serum were withdrawn, all the uneasy feelings soon returned; particularly the sense of weight; and the pain and tenderness of the abdomen.

"Two days after the tapping I find that the pain which increased after the operation, had continued ever since, and at times that it was "*excruciating.*" He compares his suffering to the scraping with wire combs. The evacuations are more jelly-like. The pain, when the intestines are acting, is at times quite intolerable, and makes him cry out. The pulse becomes quicker, and skin is dry. The burning heat at the stomach, the irregularity of the bowels, the vomiting, and the distress after eating any thing are quite as troublesome as ever."

He was again tapped, but death put a period to his sufferings on the 21st of June.

*Dissection.* Three pints of dark coloured fluid in the abdomen; the intestines appeared at once, but no omentum was visible; they were redder than usual; adhered together; and the thickened tuberculated state of the peritonæal coat was every where seen. The peritonæum lining the abdomen, shewed the diseased structure in the most perfect form. In some places the tubercles were distinct, and well defined, having patches of turgid vessels between them; in others, the tubercles having coalesced, gave to the surface a rough appearance, with protuberances of various sizes diffused over it. On searching, the omentum was found converted into a large irregular shaped tuberculated mass, occupying part of the epigastrium and left hypochondrium, bearing some resemblance to a diseased spleen. Around it the peritonæal coat of the stomach and intestines was much thickened, with great agglutination of the contiguous parts. The great curvature of the stomach was studded with tubercles; part of the arch of the colon was embedded in the diseased omental mass, which, when cut into, exhibited a firm, cheesy sort of texture; some portions shewing distinct circular tubera, corresponding with the inequalities on the outer surface. Both the mesentery and mesocolon were very much diseased, having a dense leathery appearance, and being studded with tubercles. All the glands were enlarged; the peritonæum lining the diaphragm was nearly half an inch in thickness. The liver was of a loose texture, and of a copper colour; the spleen, the kidnies, and the pancreas were natural. The thoracic viscera were sound.

In addition to his own experience, Dr. Baron has searched the writings of many authors, and has adduced various instances bearing a close analogy to the disease under consideration. In our own practice we have met with some cases of this kind, and we have recorded a short account of a dissection of one, in the 3d volume of the Medico-Chirurgical Journal, page 167, Monthly Series. The following short Extract will convey some idea of the appearances.

"On laying back the parietes of the abdomen, a bag appeared adhering firmly to the liver, spleen and diaphragm, and reaching down to the pubes. It was adherent on the sides and back; but, on raising it at the bottom, the rectum was seen descending from it into the pelvis. All within it felt a semi-solid mass. It was cut into, when the various convolutions of the intestines were found winding through a mass of mesenteric disease. Some of the mesenteric glands were as large as nutmegs. *The intestines, in every part of their track, were fixed in this diseased structure, and could not possibly have had any peristaltic motion. They maintained their place, and even their calibre, when the morbid mass was cut through in all directions. The liver was studded throughout with tubercles.*" Med. Chir. Journal for January 1817.

The above was in a child three years of age, who had been ill for several months, and the disease considered as *dropsy*. We did not see the patient alive, and were only requested to ascertain the cause of death by the parents.

In the second Chapter, Dr. Baron endeavours to trace the connexion between tubercles and hydatids, and to prove that the *former* are conversions or more advanced stages of the *latter*. In this we think that our author has been more successful, than in the attempt to ascribe the formation of the abovmentioned bodies to the absorbent instead of the sanguiferous vessels. We do not indeed say, that it is simply an *inflammatory* action of the vessels that can produce these morbid growths; but the said vessels may have many other morbid actions than those observed in the process of inflammation. At all events, we believe that the vascular, lymphatic, and nervous systems of parts are so intimately blended in structure and function, that few morbid actions or states are *exclusively* seated in one or other of them.

*Ratio Symptomatum.* The connexion between symptoms and altered texture, in the disease under consideration, is more satisfactorily traced than in most other disorders, especially when the abdomen is the seat of the complaint. A glance at the functions of the alimentary canal will explain the symptoms which arise from this source, when the very fountain of nutrition is choaked up. In the thorax or other parts the symptoms are less decided, and likewise less alarming.

Although our author has adduced strong reasons for believing that the tuberculated affections of the serous membranes do not proceed from *inflammation*, and that the disease may exist without the symptoms which point out that state; yet it is certain, that inflammation may be conjoined with it, and that it often attends it, in some stage or other of its progress.

"But when this combination takes place, the remedies which are generally successful in removing either acute or chronic inflammation, are of little avail; for even should the inflammatory symptoms be subdued, the other affection would, probably slowly, but certainly, advance to a fatal termination." P. 130.

Dr. Baron suggests, as a branch of his doctrine, that scrofula, and more especially tubercular phthisis, owe their origin to the same hydatid formation as the disease under consideration. When it attacks the membranes of the abdomen, its course is more rapidly fatal than when it occurs either in the pleura or lungs, and that for obvious reasons.

The disordered digestion and the irregular state of the bowels, with the weight and uneasiness about the abdomen, are generally to be met with at the onset, but are too often overlooked, till tumefaction, tenderness, vomiting, and all the other characteristic signs have taken place. Then too, the pyrexial symptoms become most manifest, probably depending on the supervention of inflammation. When this happens, there is an *increase* of the abdominal tenderness; heat of skin; thirst; parched tongue, &c. In some cases, effusion; in some, simple adhesion; and in others ulceration may be the result.

Although the tubercular affection often spreads along the serous membranes of the *abdomen*, without touching the parenchymatous structure of the viscera, yet it is not so in the thorax, for we seldom see the tubercles or accretions in the pleura, without detecting the *former* also in the lungs themselves.

Dr. Baron draws our attention to the resemblance between the state of the tongue of persons labouring under the tuberculous disease of the peritonæum, and those affected with diabetes. But there is more analogy than this.

"The external characters of the respective complaint are very much alike, and a diseased condition of the lungs is very common to both. The one disease seems to proceed to a fatal termination, by an imperfect assimilation, and the excretion of what ought to go to nourish the body; in the other, absorption and assimilation are nearly stopped; and it is not difficult to see how these different causes produce nearly similar results." P. 136.

The swelling of the extremities is not a constant symptom, and it is sometimes confined to one side, which is satisfactorily accounted for, either by the effect of pressure, or by the extension of the disease to the lymphatics of the limb affected.

*Diagnosis.* This our author thinks is not difficult, after the disease has actually taken place. Dr Baron here presents us with a short recapitulatory view of the prominent features of the complaint, which may enable us to contrast them with those for which they might be mistaken.

"There is, in the outward appearance of persons labouring under this complaint, an expression of distress and wretchedness which is scarcely to be described. The incessant feeling of weight and uneasiness about organs whose functions are necessary to life, is sometimes changed for a state of acute pain. The former sensation extracted, from an infant, moans of the most piteous nature;—another patient declared it to be intolerable, begging for relief, and saying that he felt as if he were about to be squeezed to death. All patients do not suffer so much from this symptom; but I have seen none who did not complain of it more or less."

With ascites it is most likely to be confounded, because fluid may be effused, and fluctuation felt. The evacuation gives of course, but a momentary relief. When there is no effusion, and the accretion of the viscera is complete, there is no difficulty in ascertaining the complaint. The belly is generally protuberant, hard, and unyielding, communicating the sensation which grasping a solid tumour would give. The great characteristic symptoms, however, arise from the extension of the disease to the alimentary canal and mesentery, for then the functions of digestion and assimilation, together with the peristaltic motion of the intestines become interfered with, and the alarming characters of the disease are developed. There are other tumefactions of the abdomen, arising from enlargement of particular viscera, as of the liver, uterus, and ovaria, which may be mistaken for the complaint in question; but the specific symptoms which appertain to these particular disorganizations, will always enable an attentive practitioner to discriminate.

In respect to *Prognosis,* it is almost invariably unfavourable. Possibly, in its earliest stages, its prog ess may be checked. Some patients live longer than others; but, in general, its duration seems to be determined by the time required to bring it to that state wherein absorption from the alimentary canal is obliterated, or nearly so. After that period, the patient lives as long as the body can feed upon itself.

*Treatment.* When the disease is established, the most powerful resources of our art become unavailing, and we are forced to see our patients pining in wretchedness, without being able to afford them even temporary relief.

We are unacquainted with the means of removing the altered structure in *this* disease. Greater disorganizations may be got rid of, by the powers of the body, in other situations; but this cannot be expected when the digestive organs, through which our agents act, form the seat of the malady.

Local and general bleeding with blisters, gave but very transient relief; no more than arose from the temporary suspension of inflammatory pains. Without free evacuations from the bowels, the distress becomes insupportable. At the same time the nausea and vomiting render it extremely inconvenient and distressing to exhibit purgative medicines. Potassa and soda will sometimes mitigate the gastric irritability; at other times the mineral acids; and the gastrodynia has been occasionally relieved by the oxyde of bismuth. Mercurial purgatives often do good, but Dr. Baron thinks that they do not afford any relief beyond what proceeds from their evacuant properties. On the other hand, the constitutional effects of mercury are injurious. Anodynes Dr. Baron has employed, in all shapes, but with very little relief to the patient. They do not abate the nausea of the stomach, while they increase the constipation of the bowels.

"In one case of physconia, connected apparently with a disease of the ovary, I found the swelling altogether removed by a solution of elaterium. It induced great, and long-continued nausea and vomiting. In another case, I think there was decided benefit obtained by the use of muriate of lime. Some other facts, shewing the disappearance of morbid growth under the influence of nausea, whether caused by medicine or other means, will hereafter be mentioned." P. 149.

Dr. Baron here makes many intesting remarks on *chronic* diseases in general, which he believes, with much justice, to be connected with that part of the constitution "on which the growth and support of the body depends." A person labouring under chronic visceral disorder, we shall find fatigued by slight exertion, while the circulation is hurried, the respiration quickened, and the nervous system agitated by circumstances that formerly did not at all excite him. His sleep is disturbed; his appetite impaired, or irregular, He is oppressed or drowsy, or feels flushed after eating; the bowels are irregular; the urine often thick and turbid. Many of these symptoms, our author observes, may frequently be discovered in young people, anterior to their falling into a much worse condition; and such maladies assuredly will follow, unless obviated by proper means. Defects in the *nutritive system* may be traced in all this; and it is at such periods, Dr. Baron thinks, that morbid changes are most apt to occur, engendering tubercles in the lungs or elsewhere, and laying the foundation for the most fatal cachectic diseases.

"A similar disposition in the system is sometimes announced by other symptoms. The skin perhaps is dry and scaly, or it may be covered with eruptions of various kinds. The eye-lids look red; the lips, especially the upper, are thicker than natural; the nose and mouth are surrounded by scabby blotches; the belly is tumid; and glandular swellings may probably be detected in the neck or other parts. These are the outward and visible signs of imperfect nutrition. They are daily seen among the ill nourished children of the poor, and frequently too in the families of the rich, when a scrofulous tendency exists. In the one case, there may be a positive deficiency of nutriment; in the other, there is a defect in the organs by which the nutriment is transmitted and assimilated. They may be obstructed altogether; or they may be gorged, and unable to dispose of properly the chyle which has been formed In this case over-feeding and pampering may produce many of the disorders that are incident to an opposite state." P. 152.

Dr. Baron illustrates this reasoning by analogies drawn from the vegetable and animal kingdoms. Thus when trees grow languidly, are covered with parasitic plants and animals; when their bark is rough, &c. we infer that the soil, the situation, or the conduits of nourishment are in fault. We therefore clear out the roots; supply manure; remove those parasites that have fastened upon the epidermis, and, if not too late, a healthy and vigorous growth results.

On the other hand, let us examine a horse out of condition. His hide is harsh and scaly, closely binding his flesh; his heels are cracked. He is emaciated; short-winded, and unfit for labour. He sweats on the least exertion, and his food passes through him undigested.

"Look at the same animal after the extreme vessels on both the surfaces have been set free, and brought into a healthy state; the internal by purging, and the external by the well applied labour of the groom. See how quickly and steadily goes on the process of digestion and assimilation; how the skin becomes soft and pliant; how the animal acquires flesh and strength, and spirit, and energy, greater far than he ever attains in his natural state of existence; how all appearances of disease vanish; and how he becomes master of those wonderful powers which astonish us in the hunter or the race horse." P. 154.

From these observations in the stable, we may turn to those who are trained for prize fighting or other extraor-feats of strength or activity. Their preparations correspond with what has been described. All impurities are eliminated; all the viscera and their emunctories are purged by an attenuating diet, laxatives, and diaphoretics.—Then, by judicious feeding, exercise, and friction, the body is brought to a state capable of enduring the most extraordinary exertions.

These facts should not be overlooked; for, surely, any physician might gain credit for bringing a poor emaciated patient, enduring and threatened with many bad symptoms, into a state of vigorous health, even should he be guided by such homely analogies as those stated by our author.

"By keeping the alimentary canal in a healthy condition; by freeing the mouths of the lacteals from obstructions; by apportioning the diet, both in quantity and quality, to the wants and powers of the system; by avoiding every thing whereby these powers may be either too much excited or exhausted; by applying suitable means to subdue such local affections as may either have existed, or may arise, in the course of the complaint; by attending closely to the state of the skin; by promoting the healthy action of its extreme vessels, by bathings of various kinds, by frictions, and well regulated exercise, we may often witness the most gratifying results, in such persons as I have alluded to, instead of being compelled to watch the progress of fatal disorganizations, which probably would have arisen, had less rational and comprehensive views directed our practice." P. 156.

In illustration of the effects of long continued nausea in exciting the action of the lymphatics to the removal of morbid growths, in various situations, Dr. Baron brings forward some very interesting facts, from the practice of Dr. Jenner, of which we shall present the reader with an analysis.

1. A gentleman with deranged lymphatic system, and affected with scirrhous tumours was sent to a warm climate for the benefit of his health. The passage was long and tempestuous; and the invalid was *sea sick*, as well as sick of the sea during the whole time. At the end of the voyage the scirrhous tumours were gone.

2. A lady of delicate organization, and strumous habit, received accidentally, a blow on the right breast, followed by some uneasiness, and the formation of a hard tumour on the superior part of the mamma. Another and another succeeded, till three fourths of the breast assumed the characteristic marks of complete scirrhosity. As it resisted all discutient means, Dr. Jenner recommended the knife; but, unwilling to undergo an operation, the young lady repaired to London, and, under the care of a surgeon, took emetic tartar, in nauseating doses, repeatedly during the day, so as not only to be sensibly, but distressingly felt, though never, if possible, to excite vomiting. She grew thin, and somewhat weak; but, as it was manifest that an amendment in the breast was observable, she persevered, till, to Dr. Jenner's astonishment, the absorbents had entirely removed the disease. She gradually desisted from the use of the medicine, and enjoyed good health for many years afterwards.

3. A young woman, 22 years of age, had removed from the country to London, and there engaged in a sedentary occupation. A cough gradually stole upon her, and increased so as to oblige her to return to Cheltenham. When Dr. Jenner saw her, "no human being could present a more correct picture of a person in an advanced stage of phthisis pulmonalis." From the general account of the symptoms, rise, and progress of the disease, Dr. Jenner could not hesitate to consider this as an unequivocally tuberculous case, and not one of inflamed mucous membranes. She recovered under the *sickening plan*, which she went through with great resolution. The medicines employed for this purpose were tincture of squills, and the tincture of digitalis, three parts of the former to one of the latter; beginning with twenty drops, ter die; but the stomach becoming irritable, five drops at last were a sufficient dose.

Dr. Jenner thinks that, on a retrospective view of the most popular remedies hitherto employed in these pitiable maladies, they will be found to consist of such things as produce a *nauseating* effect on the stomach, either from immediate contact, or secondarily through their influence on the brain.

"As we have been for ages, says Dr. Jenner, in total darkness respecting the means of removing tubercles from an internal organ, when formed to any extent, may we not hope, that the little glimmering which this seems to let in upon us, may lead to something beneficial to humanity; and, without putting theory to the rack, may it not be supposed that the stomach and intestines, being thus deranged in their ordinary functions, and the supplies for the absorbents becoming scanty and deteriorated, they the more readily seize on bodies, not those only that are absolutely extraneous, but which come so near that point, as tubercles." P. 162.

PART II. *Accretions of the Serous Membranes of the Chest.*

We have bestowed such labour in conveying a clear analysis of the *first* part of this work to our readers, that the *second* may be compressed into much less space.

Our author observes, that we must distinguish between simple adhesion of the pleura, resulting from inflammatory action, and the granular or tuberculated disease of the said membrane; an affection connected with a disorder of much more serious and extensive nature. The symptoms attendant on such affections, as far as Dr. Baron has been able to judge, are a dull obtuse pain, or rather a sense of weight or tightness about the chest; sometimes pain about the scapulæ and clavicles; quick respiration; short cough, without expectoration. As the disease advances, and the accretions of the pleura are complete, the countenance becomes anxious and sallow; the shoulders are drawn forward; the ribs do not heave freely, but the whole chest seems to rise at once, with great action of the muscles of the trunk; pulse variable, but easily accelerated by the slightest exertion. When the disease has proceeded far, with much alteration of texture in the pleura or lungs themselves, then dissolution generally takes place suddenly. The effusions which occasionally are found in this complaint are of the same nature as those in the abdomen, and the result of inflammatory attacks supervening on the original disease. The following case, which is somewhat condensed, will illustrate the foregoing symptomatology.

"Leonard Edwards, æt. 15, affected with frequent dry cough, laborious respiration, sense of tightness and oppression at the epigastrium; nights restless; morning perspirations; tongue white; appetite impaired; pulse 100; countenance sallow; languor and weakness; shoulders prominent; body bent forward; cannot elevate the chest, nor throw the body into an erect position, without great distress. Has been ailing five weeks; complained at the first of pains about the thorax. Blister to the thorax; purgatives of decoct. aloes c.—half an ounce of the mist. ferri compos. bis die.

"Three days afterwards the cough and difficulty of breathing were reported to be so much relieved, that he walked up and down stairs, and in the yard of the infirmary. Still complained of sleeping very ill; an anodyne draught was given at bed time. He took his supper and went to bed. At one o'clock in the morning he suddenly expired.

"*Dissection.* On cutting into the belly, the peritonæum, where it covers the diaphragm, the sides of the abdomen, and the convex surface of the liver, instead of being smooth and shining, was much thickened, and had a rough granulated appearance. The other organs of this region sound. On elevating the sternum, all the thoracic viscera seemed to be united into one mass, without distinction of parts. The pleura costalis and pulmonalis universally adherent, and converted into a dense rough cartilaginous-looking substance, which near the diaphragm was nearly half an inch in thickness. It was easily separable into layers, and numberless small tubercles were seen between each. The lungs themselves were turgid with blood, but otherwise healthy. The outer surface of the pericardium partook strongly of the disease of the pleura. The inner was sound. Right auricle of the heart greatly dilated; brain and its membranes sound." P. 174.

Dr. Baron next relates some particulars of two cases where the disease had extended from the pleura to the lungs themselves, with remarkable disorganization of the latter; and then introduces a long history "*atrocis rarissimique morbi*," which is related by Boerhaave, and he conceives bears a close affinity to the disease under consideration. Numerous other cases are also related or quoted, all bearing upon the same point.

Into the second chapter of this division of the work, entitled, "Further observations illustrating the connection between hydatids and tubercles," we cannot enter, since it would be impossible to give any connected view of our author's arguments. We must therefore refer to the work itself, those who are interested in the decision.

In the third chapter our author relates some interesting cases of other tubercular disorganizations of the thoracic and abdominal viscera, with remarks on vomica, of which we shall take some brief notice. In the first case there was accretion of the aorta and œsophagus, with ulceration and stricture of the latter, and disease of the cardia. In the second, there was accretion of the aorta to a diseased pancreas; and in the third, there were tubercles hanging in clusters from the valves in the left side of the heart. We shall give an outline of the second and third cases.

J. P ætat 53, March 15. Liver enlarged, and can be felt considerably below the false ribs; violent pulsation in epigastrio; sense of weight and pain about the right side; uneasiness and soreness about the shoulders, clavicle, and neck, with distressing restlessness. Lies chiefly on his left side and back; eyes and skin tinged yellow; appetite impaired; pulse 80; urine remarkably high-coloured, staining linen, but not depositing a sediment.

The complaint began six months before, with symptoms of dyspepsia, which were relieved by the usual means. But by irregular living, he got worse, when he was directed to take the blue pill and aloes, under which he so far recovered, that, on the 11th of January, he was able to use violent exercise in dancing for a whole evening. This did him harm— he was sent to Cheltenham, and took mercury in all forms, yet the disease increased He lost flesh rapidly, and was obliged to take large quantities of opium to procure him rest. The beating at the epigastrium attracted a great deal of his own notice, and harrassed him much. He had a most distressing sense of heat at the stomach, which was effectually relieved by the oxide of bismuth. The disease resisted every remedy, and proved fatal on the 15th of April.

*Dissection*. Liver much enlarged, and studded with irregularly shaped tubercles, some of them very hard, and almost cartilaginous. The interstitial substance seemed healthy. The ducts of the gall bladder were thickened and compressed between the hard and dense parts of the diseased liver, and an enlarged and tuberculated pancreas. The *latter* had formed a close and strong adhesion with the abdominal aorta, and its pulsations were transmitted through the medium of the diseased mass to the epigastrium. P. 286.

The third case was that of a woman, 29 years of age, who became an out-patient of the Infirmary early in 1815. She had laboured under amenorrhœa, and complained of pain and uneasiness of the head, shortness of breath, fluttering about the heart, constipation, languor, and depression. She had a peculiar appearance of anxiety and distress in her countenance, which the symptoms did not well account for. She was treated with purgatives and tonics; the symptoms were somewhat relieved, but by no means effectually. In March she was seized with purpura, which harrased her more or less till she died. She was bled repeatedly and purged, with temporary benefit. She had, at intervals, rigors, followed by pain of the chest, and great difficulty of breathing, the fluttering at the heart being a constant subject of complaint. The uneasiness about the head also increased, and the action of the carotids was so violent as to attract much of her own notice. She now and then fell into a stupor, from which she generally awoke in a state of wildness and amazement. She had also a violent cough, but without expectoration. Pulse about 120, hard and bounding till the last, but never irregular. The night before she died, she complained of great coldness of the extremities. She went to sleep as usual, but died at eleven the next day.

*Dissection.* No marks of disease any where but in the heart. It was of a larger size than natural, and about ten ounces of fluid were found within the pericardium. The capacity of the right auricle was increased greatly. From the mitral and semilunar valves, small tuberculated fringes hung into the cavities; and in the ventricle, they were likewise attached to the columnæ carneæ. These tubercles were very distinct, about the size of millet seed, and resembled precisely those which our author had seen on the peritoneum.

Our closing limits will not permit us to do justice to Dr. Baron's interesting remarks on vomica. In patients under this affection, he has found a short frequent cough, with little expectoration; respiration easily hurried on motion; pulse readily accelerated; obtuse pain or uneasiness in some part of the chest. Sometimes head-ache is very distressing. People in this condition, unwilling to be thought ill, will sometimes talk loudly, and breathe deeply apparently with ease. They generally, however, lose flesh; the face becomes pallid and unhealthy looking, with feelings of languor and distress. After a time, the patient is perhaps surprized by the expectoration of a large quantity of purulent-looking matter, after an unusual fit of coughing. He may either be suffocated at once, or he may linger for some time; but death is generally the result.

" In none of these cases that I have alluded to above, had there been any attack of pneumonia; and every pathological work contains accounts of cases where " *bags of matter*" have been found within the lungs and other viscera, even where no symptoms of inflammation previously had existed." P. 295.

Dr. Baron is therefore of opinion, that matter, or that which resembles matter, may be formed independently of the process of inflammation, that is, through the medium of the absorbent vessels, and that many cases of what are termed vomica, are hydatids in a certain stage of their progress.

" It is enough for my present purpose, to show that hydatids, in one state of their existence, do contain such fluids as are found in vomica; and that the phenomena of the disease correspond in every point with those which sometimes attend the growth and progress of these bodies." P. 301.

Of the general merits of Dr. Baron's work, we need not express our opinion in symbol, when an inference is so deducible from the substance. It has been our object in this instance, and it will be our aim in most others, to lay before the reader a connected view and a faithful representation of the facts and opinions under discussion; leaving to himself the free exercise of his faculties, and the full prerogative of his judgment. The utility of this plan, we believe, *cannot,* and the execution of it, we hope, *shall not* be surpassed in any other publication.

> Qui cupit optatam cursu contingere metam
> Multa fert facitque.

